# Haematological and biochemical pathology markers for a predictive model for ITU admission and death from COVID‐19: A retrospective study

**DOI:** 10.1002/jha2.529

**Published:** 2022-07-12

**Authors:** Anuja Rasarathnam, Terrence Haynes‐Smith, Wassif S. Wassif, Michael S. Dodd

**Affiliations:** ^1^ Haematology and Blood Transfusion Bedfordshire Hospitals NHS Foundation Trust Bedford UK; ^2^ School of Life Sciences Faculty of Health and Life Sciences Coventry University Coventry UK; ^3^ Clinical Biochemistry Bedfordshire Hospitals NHS Foundation Trust Bedford UK; ^4^ Research Centre for Sport Exercise and Life Sciences Institute of Health and Wellbeing Coventry University Coventry UK

**Keywords:** biomarkers, COVID‐19, hyperkalaemia, ITU, NLR

## Abstract

Coronavirus disease (COVID‐19) caused by SARS‐CoV‐2 has affected over 227 countries. Changes in haematological and biochemical characteristics in patients with COVID‐19 are emerging as important features of the disease. This study aims to identify the pathological findings of COVID‐19 patients at Bedford Hospital by analysing laboratory parameters that were identified as significant potential markers of COVID‐19. Patients who were admitted to Bedford Hospital from March–July 2020 and had a positive swab for COVID were selected for this study. Clinical and laboratory data were collected using ICE system. Multiple haematological and biochemistry biomarkers were analysed using univariate and multivariate logistic regression to predict intensive therapy unit (ITU) admission and/or survival based on admission tests. Neutrophil‐to‐lymphocyte ratio (NLR) and C‐reactive protein were elevated in most patients, irrespective of ITU status, representing a common outcome of COVID‐19. This was driven by lymphopenia in 80% and neutrophilia in 42% of all patients. Multivariate logistic regression identified an increase in mortality associated with greater age, elevated NLR, alkaline phosphatase activity and hyperkalaemia. With the area under the receiver operating characteristic (ROC) curve of 0.706 +/− 0.04117, negative predictive value (NPV) 66.7% and positive predictive value (PPV) 64.9%. Analysis also revealed an association between increases in serum albumin and potassium concentrations and decreases in serum calcium, sodium and in prothrombin time, with admission to ITU. The area under the ROC curve of 0.8162 +/− 0.0403, NPV 63.3% and PPV 80.5%. These data suggest that using admission (within 4 days) measurements for haematological and biochemical markers, that we are able to predict outcome, whether that is survival or ITU admission.

## 1 INTRODUCTION

On 31 December 2019, Wuhan Municipal Health Commission, China, reported a cluster of pneumonia cases of unknown aetiology to the World Health Organisation (WHO) [1, 2]. Severe acute respiratory syndrome coronavirus 2 (SARS‐CoV‐2) was identified as the causative agent of this infection [3]. WHO director declared the SARS‐CoV‐2 as a public health emergency of international concern on 30 January 2020, which is the WHO's highest level of alarm regarding the emergence of the new epidemic viral disease. WHO announced the viral disease caused by SARS‐CoV‐2 would be named coronavirus disease 2019 (COVID 2019), and a pandemic state was declared on 11 March 2020.

Coronaviruses (CoVs) are a large viruses group belonging to the Coronaviridae family, which causes severe respiratory disease called COVID‐19 [4]. The clinical presentation of symptomatic patients of COVID‐19 is fever, which is defined as an axillary temperature of 37.5°C or higher, dry cough, shortness of breath, dyspnoea, loss of smell and/or taste, fatigue, muscle pain and pneumonia [5, 6]. Respiratory droplets and human‐to‐human contact are the main routes of transmission of the virus [3, 7].

Symptomatic patients can require hospitalisation due to the acceleration of the infection and may subsequently require admission to an intensive therapy unit (ITU). A minority of patients develop severe pneumonia, acute respiratory distress syndrome, acute respiratory failure, refractory metabolic acidosis, coagulopathy, septic shock, multiple organ failure and consequently death [3, 8–11]. Patients who are above the age of 65 with previous co‐morbidities such as diabetes, cancer, cardiovascular diseases, respiratory disease, leukaemia and myelodysplastic syndrome are more likely to develop serious clinical complications, and they constitute between 50% and 70% of deaths [12, 13].

As of 24 January 2022, 228 countries across the world have positive COVID cases: 349,641,119 confirmed cases and 5,592,266 deaths worldwide (2). Whilst in the United Kingdom there were 15,859,292 positive cases and 153,862 deaths within 28 days of a positive test. The first COVID positive patient was admitted to Bedford Hospital on 21 March 2020, although a few cases were reported earlier in other regions. This retrospective study aims to investigate the pathological findings of COVID‐19 patients at Bedford Hospital by analysing haematological and biochemical laboratory parameters that were identified as potential significant markers of COVID‐19.

We hypothesized that using admission data and multivariate logistic analysis that we could develop a predictive model for either survival or ITU status from COVID. The aim of this study was to identify the key variables, which significantly varied using univariate analysis and that would allow predictions of outcome.

## 2 METHODS

Retrospective data were collected between 21 March 2020 and 19 July 2020, which has been classified as the first wave at Bedford Hospital. Data have been statistically analysed to assess any emergence of specific patterns at Bedford Hospital as there was a lack of specific therapies or clear strategies to treat COVID‐19 at the time. Hence, these data reflect an accurate indication of the severity of COVID‐19. The list of biomarkers investigated in this study is platelets, mean corpuscular haemoglobin concentration (MCHC), lymphocytes, neutrophils, neutrophil to lymphocyte ratio (NLR), prothrombin time (PT), activated partial thromboplastin time (APTT), C‐reactive protein (CRP), calcium, albumin, alanine aminotransferase (ALT), alkaline phosphatase (ALP), urea, glucose, total bilirubin, potassium, sodium and creatinine. The biomarkers studied have shown promise in other studies to predict the morbidity and mortality of COVID‐19 in patients who are hospitalised [1, 4, 6, 11, 14, 15].

### 2.1 Study subjects

According to the interim guidance from WHO, patients’ throat swab is tested using real‐time reverse transcriptase‐polymerase chain reaction, which detects the presence of SARS‐CoV‐2 RNA, which are classed as COVID‐19 positive. One hundred and fifty eight patients, aged 26–102 years (mean age 73.34 ± 14.49 years), were recruited retrospectively for the study. All patients who were included in the study had a positive swab result on admission to Accident and Emergency. They comprised 112 (71%) males and 46 (29) females. The patients were categorised into four groups in terms of severity and the outcome of the disease. Permission to conduct the study was approved by the Ethics Committee of Coventry University (#P111791).

### 2.2 Clinical laboratory data

Clinical and laboratory data required for the study were collected during routine clinical consultations and using Integrated Clinical Environment (ICE), which is Bedford Hospital's Pathology reporting system. Blood was collected within 4 days of admission to Bedford Hospital. The following analytes and results were extracted from the Bedford Hospital ICE database: platelets, MCHC, lymphocyte, neutrophils, NLR, APTT, prothrombin time, CRP, adjusted calcium, albumin, ALT, ALP, urea, glucose, total bilirubin, potassium, sodium and creatinine.

### 2.3 Statistical analysis

The parameters were expressed as median with IQR. Differences in the parameters between groups were determined by an independent *t*‐test for normally distributed data, and Mann–Whitney *U* test was used for non‐normally distributed data. Two‐way ANOVA was used to compare survival within the non‐ITU and ITU groups of patients. Gender was compared using chi‐squared test.

Univariate and multivariate logistic analysis (Graphpad Prism 9) was performed on admission haematology and biochemical results to generate a model and determine whether individual or combination of analytes was able to predict either ITU admission or mortality. Analytes from the univariate analysis, which had a *p* value <0.1 in the likelihood ratio test and area under the ROC curve (AUC), were used in the multivariate logistic regression analysis along with gender and age. ROC analysis was performed, and AUC, positive predictive and negative predictive power were calculated. Both Hosmer‐Lemeshow and Log‐likelihood ratio (G squared) were used to assess goodness of fit for the logistic regression models used.

## 3 RESULTS

**FIGURE 1:**
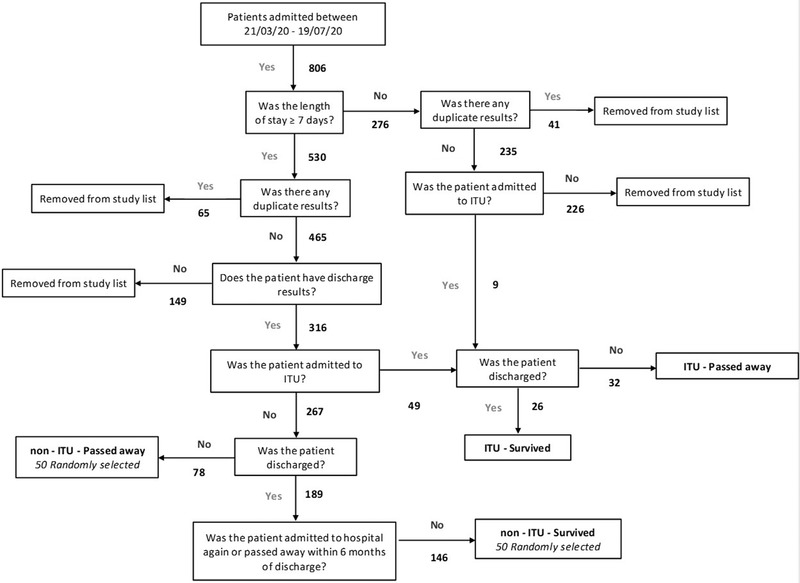
Study flow chart, showing how patient selection was achieved from the Integrated Clinical Environment (ICE) database at Bedford Hospital. Eight hundred six records were found for admission to Bedford Hospital trust with a positive polymerase chain reaction (PCR) test for severe acute respiratory syndrome coronavirus 2 (SARS‐Cov‐2) between 21/03/20 and 19/07/20. One hundred fifty‐eight patients were selected for our study, who matched our criteria and fitted into either survived or passed away and Intensive Therapy Unit (ITU) or non‐ITU

In this study we investigated patients that were admitted with a positive polymerase chain reaction (PCR) test for SARS‐Cov‐2 between 21/03/20 and 19/07/20 into Bedford Hospital trust; this resulted in 806 records. Figure 1 shows the PRISMA diagram generated to classify patients into non‐ITU/ITU and survived/non‐survivors. From these 806, 58 (7%) patients were admitted to ITU, 26 (45%) were discharged (ITU – survived), and 32 (55%) passed away (ITU – passed away). Again from these 806, 267 (33%) met our criteria and were not admitted to ITU; of these 78 (29%) passed away, and 50 were randomly selected for inclusion in our study (non‐ITU – passed away). Of the 189 that survived, 146 (77%) were not readmitted or passed away 6 months post‐discharge; of these 50 were randomly selected for inclusion in our study (non‐ITU – survived). Clinical characteristics are presented in Table 1. In the non‐ITU group, patients who passed away were significantly older (median age 82 [75–90], *p* < 0.01). There was no significant difference in the length of stay between survived and passed away within the non‐ITU group. For ITU, whilst there was no significant difference in age between survived and passed away groups, patients in ITU were significantly younger than those not in ITU (*p* < 0.01). Whilst men made up the largest proportion of patients in our study with SARS‐Cov2, there was not a significant difference in survival compared to women, irrespective of ITU status.

**TABLE 1:** Clinical, biochemical, and haematological characteristics of the four study cohorts

		Non‐ITU							ITU								
		Survived			Passed away				Survived				Passed away				
	Units	Median	LQR	UQR	Median	LQR	UQR	Survival p value	Median	LQR	UQR	p value compared to Non‐ITU	Median	LQR	UQR	p value compared to Non‐ITU	Survival *p*‐value
Age	Years	76	62.75	82.25	82	74.75	90.25	*<0.01*	60	51	66.5	*<0.001*	65	59	75.5	*<0.001*	*0.06*
Gender ‐ female		38%			28%				23%				22%				
Length of stay	Days	16	11.75	31	16	10	22.25	*0.47*	28	13	39.25	*0.08*	11	6	15	*0.25*	*<0.001*
Platelets	10ˆ9/L	229.5	161.25	305.25	210.5	163.5	319.5	*0.99*	217	157	304.75	*0.98*	219.5	189.75	292	*0.99*	*0.93*
MCHC	g/L	360	341.5	373	349	338	357.25	*0.29*	357	343	366.5	*>0.99*	357	344	364	*0.66*	*0.99*
Lymphocyte	10ˆ9/L	1.10	0.70	1.40	0.80	0.58	1.22	*0.12*	0.90	0.68	1.20	*0.55*	1.00	0.60	1.30	*0.73*	*0.97*
Neutrophils	10ˆ9/L	6.35	3.88	8.93	7.75	4.68	11.40	*0.87*	7.80	4.65	13.10	*0.31*	7.35	4.65	10.88	*0.27*	*0.81*
NLR		6.24	3.50	8.72	10.92	5.63	16.96	*<0.01*	8.22	5.72	16.07	*0.42*	8.51	5.15	12.31	*0.14*	*0.86*
aPTT	Secs	28	25	32	29.5	26.75	36.25	*0.86*	26	23.75	31	*0.84*	29	25.75	33	*0.66*	*0.99*
Prothrombin time	Secs	12.00	11.00	14.00	12.00	11.75	15.00	*0.63*	12.00	11.00	13.00	*0.47*	11.50	11.00	13.25	*0.99*	*>0.99*
CRP	mg/L	62.00	17.75	155.75	81.00	48.75	126.50	*0.97*	81.00	49.20	172.50	*0.99*	131.00	47.25	241.50	*0.16*	*0.63*
Adjusted calcium	mmol/L	2.30	2.17	2.39	2.31	2.25	2.38	*0.73*	2.26	2.15	2.42	*0.96*	2.23	2.16	2.33	*0.06*	*0.83*
Albumin	g/L	34.00	29.75	39.00	30.00	27.75	35.00	*0.08*	36.50	32.75	40.50	*0.19*	37.00	33.25	40.00	*<0.001*	*>0.99*
ALT	IU/L	24	15	39	24	13	34	*0.99*	42.00	26.50	59.50	*0.24*	26.5	17	51.25	*0.84*	*0.62*
ALP	IU/L	73.00	51.25	93.25	97.50	72.00	138.25	*<0.05*	82.00	59.00	138.00	*0.93*	79.00	61.50	116.50	*0.13*	*0.99*
Urea	mmol/L	5.65	3.65	9.13	8.15	4.90	12.10	*0.18*	7.40	5.40	13.00	*0.67*	6.40	5.15	12.10	*0.81*	*0.67*
Glucose	mmol/L	6.30	4.98	7.78	6.90	5.60	8.70	*0.67*	8.10	5.80	11.70	*0.40*	6.80	6.00	10.50	*0.98*	*>0.99*
Total bilirubin	μmol/L	9.00	7.00	13.00	10.00	6.00	14.00	*0.93*	11.50	7.75	14.00	*0.43*	8.00	6.00	14.75	*0.93*	*0.46*
Potassium	mmol/L	4.0	3.58	4.30	4.0	3.70	4.63	*0.31*	4.3	3.75	4.55	*0.41*	4.1	3.90	4.45	*0.55*	*0.76*
Sodium	mmol/L	137.5	135.00	140.00	140.0	136.75	143.00	*0.16*	137.5	134.00	141.00	*0.99*	136.0	133.25	138.75	*<0.001*	*0.79*
Creatinine	μmol/L	81.50	72.00	98.50	94.00	70.50	128.00	*0.79*	88.50	68.75	120.75	*0.47*	96.50	78.00	122.25	*>0.99*	*0.92*

*Note*: To compare the survivors and non‐survivors of non‐ITU and ITU groups of patients’ two‐way ANOVA was used. Gender was compared using chi‐squared test. *p* < 0.05 is considered significant.

Abbreviations: ALP, alkaline phosphatase; ALT, alanine aminotransferase; aPTT, activated partial thromboplastin time; CRP, C‐reactive protein; MCHC, mean corpuscular haemoglobin concentration; NLR, neutrophil to lymphocyte ratio.

The clinical, biochemical and haematological characteristics of the four groups are presented in Table 1. In the non‐ITU group, NLR (10.92 [5.63–16.96] *p* < 0.01) and ALP (97.50 [72.00–138.25] *p* < 0.05) were elevated in the group that passed away. In the ITU group, there was no significant difference between the survived and passed away groups. The patients in ITU were significantly younger than those in the non‐ITU group, between the survived group and the passed away group. Those that passed away in ITU, versus non‐ITU, showed significantly elevated albumin (37.00 [33.25–40.00] *p* < 0.05) and lower sodium (136.0 [133.25–138.75] *p* < 0.05). Hyponatraemia (<135 mmol/L) was seen in 25% of non‐ITU and 38% of ITU patients. Lymphopenia (<1.5 × 10ˆ9/L) was seen in 80% of non‐ITU and 81% in ITU patients and neutrophilia (>7.5 × 10ˆ9) in 44% of non‐ITU and 41% of ITU patients.

### 3.1 Modelling survival

**FIGURE 2:**
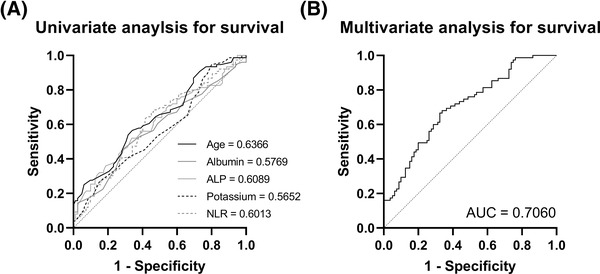
(A) Receiver operating characteristic (ROC) analysis showing univariate logistic regression analysis, which was performed to determine if admission data could distinguish survival status independent of intensive therapy unit (ITU) status. It was revealed that age, neutrophil‐to‐lymphocyte ratio (NLR), alkaline phosphatase (ALP), and potassium were significantly associated with increased survival (area under the ROC values are in the figure). (B) ROC analysis curves showing multivariate logistic regression using combined data from the univariate analysis. This revealed a significant area under the ROC curve was 0.706 +/− 0.04117 (*p* < 0.001)

Univariate logistic regression analysis was performed to distinguish survival status independent of ITU status. It was revealed that age (odds ratio [OR] 0.9624 [95% CI: 0.9389–0.9849] *p* < 0.001), NLR (0.958 [0.9143–0.9966], *p* < 0.05), ALP (0.9953 [0.9896–0.9996] *p* < 0.05) and potassium (0.5711 [0.3215–0.9723]) were significantly associated with increased survival (Table 2 and Figure 2A). Using parameters from the univariate analysis, which had a *p* value <0.1, we were able to generate a model of survival using multivariate logistic regression analysis (Table 3). The AUC was 0.706 +/− 0.04117 (*p* < 0.001 – Figure 2B), the model had a negative predictive power of 66.67%, a positive predictive power of 64.86%, and an accuracy of 65.8%. This analysis further revealed that hyperkaliaemia, along with being an older male, raised NLR and ALP, whilst lower albumin was linked to decreased survival.

**TABLE 2:** Univariate logistic analysis of survival

	Odds ratio	95% CI	*p* (likelihood ratio test)
Age (years)	0.9624	0.9389–0.9849	0.001
Platelets	0.9994	0.9969–1.002	0.3016
MCHC	1.015	0.9963–1.035	0.1165
Lymphocyte	1.533	0.8892–2.782	0.1261
Neutrophils	0.9696	0.9070–1.033	0.3422
NLR	0.958	0.9143–0.9966	0.032
aPTT	0.9857	0.9465–1.021	0.4264
Prothrombin time	1.011	0.9889–1.053	0.346
CRP	0.9992	0.9960–1.002	0.6356
Adjusted calcium	0.7894	0.06667–9.107	0.8491
Albumin	1.047	0.9943–1.106	0.0813
ALT	1.002	0.9973–1.009	0.3788
ALP	0.9953	0.9896–0.9996	0.031
Urea	0.959	0.9031–1.013	0.1332
Glucose	0.9682	0.8910–1.043	0.4009
Total bilirubin	1.008	0.9677–1.053	0.6827
Potassium	0.5711	0.3215–0.9723	0.0388
Sodium	0.9717	0.9148–1.030	0.3332
Creatinine	0.9991	0.9943–1.004	0.6795

*Note*: Odds ratio above 1 means an increase in survival.

Abbreviations: ALP, alkaline phosphatase; ALT, alanine aminotransferase; aPTT, activated partial thromboplastin time; CI, confidence intervals; CRP, C‐reactive protein; MCHC, mean corpuscular haemoglobin concentration; NLR, neutrophil to lymphocyte ratio.

**TABLE 3:** Multivariate logistic analysis parameters for survival

	Odds ratio	95% CI	*p* (likelihood ratio test)
Gender (F)	1.561	0.7175–3.455	0.2645
Age (years)	0.9588	0.9322–0.9842	0.0023
NLR	0.9629	0.9166–1.003	0.0972
Albumin	1.016	0.9571–1.078	0.6022
ALP	0.9963	0.9902–1.001	0.1677
Potassium	0.5669	0.2967–1.023	0.0705

*Note*: Odds ratio above 1 means an increase in survival.

Abbreviations: ALP, alkaline phosphatase; CIs, confidence intervals; Gender (F), female; NLR, neutrophil to lymphocyte ratio.

### 3.2 Modelling risk of admission to ITU

**FIGURE 3:**
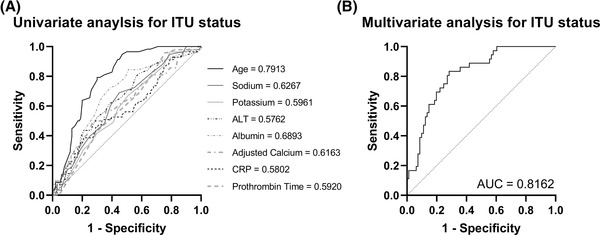
(A) Receiver operating characteristic (ROC) analysis curves showing univariate logistic regression analysis, which was performed to determine if admission data could distinguish intensive therapy unit (ITU) status. It was revealed that age, sodium, potassium, alanine aminotransferase (ALT), albumin, adjusted calcium, C‐reactive protein (CRP), and prothrombin time were significantly associated with being in ITU (area under the ROC values are in the figure). (B) Multivariate logistic analysis was performed using the univariate parameters to generate an ROC for analysing survival based on our markers. The area under the ROC was 0.8162 +/− 0.0403 (*p* < 0.001)

Along with survival, we aimed to investigate the factors that could predict ITU status based on initial admission measurements; these were independent of the clinical markers that would normally guide admittance to ITU. Univariate logistic analysis revealed that age (0.9205 [0.8911–0.9475] *p* < 0.001), adjusted calcium (0.04211 [0.002024–0.6830] *p* < 0.05), albumin (1.127 [1.061–1.203] *p* < 0.001), potassium (1.776 [1.034–3.165] *p* < 0.05) and sodium (0.9223 [0.8603–0.9830] *p* < 0.05) were significantly associated with being in ITU (Table 4). Again using parameters that had a *p* value <0.1 in the univariate analysis, we generated a model that looked for an association between admission data and admission to ITU (Table 5). The area under the ROC was 0.8169 +/− 0.0403 (*p* < 0.001 – Figure 3) with a negative predictive power of 63.33%, a positive predictive value (PPV) of 80.46% and an accuracy of 75.2%. This analysis again revealed that being male increased the chances of being in ITU, but that ITU was associated with younger patients. Increased serum albumin and potassium, whilst a decrease in PT, adjusted calcium, ALT and sodium concentrations were associated with a greater risk of being admitted to ITU.

**TABLE 4:** Univariate logistic analysis for ITU status

	Odds ratio	95% CI	*p* (likelihood ratio test)
Age (years)	0.9205	0.8911–0.9475	<0.001
Platelets	0.9998	0.9972–1.002	0.9057
MCHC	1.007	0.9880–1.027	0.467
Lymphocyte	0.9379	0.5227–1.622	0.8208
Neutrophils	1.022	0.9575–1.091	0.5028
NLR	0.9923	0.9529–1.028	0.6799
APTT	0.9676	0.9159–1.009	0.1376
Prothrombin time	0.9264	0.8029–1.001	0.0586
CRP	1.003	0.9996–1.006	0.0806
Adjusted calcium	0.04211	0.002024–0.6830	0.0252
Albumin	1.127	1.061–1.203	0.0001
ALT	1.005	0.9998–1.013	0.061
ALP	0.9976	0.9922–1.001	0.2453
Urea	1.006	0.9497–1.062	0.8384
Glucose	1.055	0.9786–1.144	0.1627
Total bilirubin	1.012	0.9698–1.057	0.5765
Potassium	1.776	1.034–3.165	0.0372
Sodium	0.9223	0.8603–0.9830	0.0122
Creatinine	1.002	0.9977–1.007	0.3159

*Note*: Odds ratio above 1 means less likely to be admitted to ITU.

Abbreviations: ALP, alkaline phosphatase; ALT, alanine aminotransferase; aPTT, activated partial thromboplastin time; CI, confidence intervals; CRP, C‐reactive protein; MCHC, mean corpuscular haemoglobin concentration; NLR, neutrophil to lymphocyte ratio.

**TABLE 5:** Multivariate logistic analysis for ITU status

	Odds ratio	95% CI	*p* (likelihood ratio test)
Gender (F)	1.363	0.4371–4.516	0.5979
Age (years)	1.074	1.035–1.120	0.0004
Prothrombin time	1.025	0.9754–1.180	0.6079
CRP	0.9968	0.9922–1.001	0.1649
Adjusted calcium	1.29	0.03278–66.00	0.8945
Albumin	0.9576	0.8755–1.044	0.3304
ALT	1	0.9907–1.011	0.9292
Potassium	0.4328	0.1856–0.9059	0.0355
Sodium	1.002	0.9022–1.111	0.9748

*Note*: Odds ratio above 1 means less likely to be admitted to ITU.

Abbreviations: ALT, alanine aminotransferase; CIs, confidence intervals; CRP, C‐reactive protein; Gender (F), female.

## 4 DISCUSSION

As of January 2022, SARS‐Cov‐2 has infected close to 350 million people worldwide and led to 5,592,266 deaths worldwide (2). Whilst more than 2 years since the original discovery of the COVID‐19, the ability to predict the outcome of patients admitted to hospital is still difficult to determine. In this study, we have performed a retrospective analysis on patients admitted to Bedford Hospital in the United Kingdom, during the initial wave in March–July 2020. Using both univariate and multivariate logistic analysis, we have been able to generate a model for predicting possible outcomes in this cohort. These include survival or admission into ITU. We have identified that increases in serum albumin and potassium were associated with the need for admission into ITU. This was coupled with a decrease in serum calcium and sodium and in PT. For ITU status, the AUC was 0.8162 +/− 0.0403, NPV 63.3% and PPV 80.5%. Whilst an increase in mortality was associated with greater age, elevated NLR, ALP and hyperkalaemia. With the AUC being 0.706 +/− 0.04117, NPV 66.7% and PPV 64.9%.

In all cohorts admitted to Bedford Hospital, there was elevated NLR (0.78–3.53 in the adult population [16]) and elevated CRP (>5 mg/L). This increased NLR was driven by lymphopenia (<1.5 × 10ˆ9/L) in 80% of non‐ITU and 81% in ITU patients and neutrophilia (>7.5 × 10ˆ9) in 44% of non‐ITU and 41% of ITU patients. Elevated CRP, NLR, lymphopenia and neutrophilia are linked to systemic infections and the development of pneumonia, all common outcomes from infection with SARS‐Cov2 [14, 17–19]. A further indication of severe pneumonia in these patients come from Wendel Garcia and colleagues (2020) who demonstrated that increased potassium was associated with a great chance of death in ITU patients [20]. Furthermore, Ravioli and colleagues demonstrated that hyponatraemia and hyperkaliaemia are related to an increase in admission to ITU and death, due to community‐acquired pneumonia [21]. Therefore these risk factors might be an indication of the development of pneumonia or an indication of the extent of damage by COVID‐19. Both of these studies are consistent with our modelling for survival and ITU admissions, which both pointed to higher serum potassium as a risk factor for ITU admission and death.

On their own, most of the biomarkers recorded in this study are unable to significantly differentiate between the four different cohorts. The power of the multivariate logistic analysis is that it pools data on multiple different risk factors and can better differentiate groups and potentially predict outcomes, whether that is survival or ITU status. In the future, the addition of further clinical markers, such as oxygen saturation, heart rate, etc., would further improve this multivariate analysis and drive a great predictive potential.

### 4.1 Limitations of our study

Logistic analysis revealed that if you were younger, you were more likely to end up in ITU. This however might be influenced by the fact that there is a greater age of patients in the non‐ITU group who scored high on the clinical frailty scale or passed away, meaning that they did not meet the criteria to be admitted to ITU or died before being transferred to ITU. Another limitation of this study is that this is only carried out in a single hospital setting during the first wave, and there is a lack of a control group to compare to. This is due to the rapid emerging nature of the first wave, and future studies would use equally weighted groups, along with a control group for comparison of haematological and biochemical parameters. Our data here were based on blood tests from within the first 4 days of admissions, where testing within the first day might provide more reliable indicators of disease. Furthermore, due to the nature of COVID‐19 infections, there was a slight bias towards more men within the study; this means it is more difficult to generalise specifically to both sexes. Future analysis will require more patient data inclusion and a need for more than one centre. Other future analysis will have to take into account whether quicker testing would have altered the results, and whether the predictive effects are equally useful for both sexes. All of these points would help to demonstrate the effectiveness of the model in its predictions and the ability to predict COVID‐19 outcomes.

## 5 CONCLUSION

It is vital in the ongoing COVID‐19 pandemic that there is an ability to tightly monitor patients that are admitted and predict the chance that they will die or need ITU. Using multivariate logistic analysis we have developed a model for predicting outcomes. Whilst differences in severity and mode of cellular damage might be different with various strains, the findings in the study underpin the underlying damage to the lungs and possible pneumonia, from COVID‐19. In November and December 2021, The UK approved two new therapeutics targeted towards COVID‐19: 1) RNA‐dependent RNA polymerase inhibitor: molnupiravir and 2) neutralizing antibody: sotrovimab [22, 23]. The effectiveness of both of these new therapeutics is in delivery to high‐risk patients to maximise survival, whilst delivering cost‐effective treatments. Our model could allow stratification into patients that would most benefit from these treatments, highlighting at‐risk patients, based on admission haematological and biochemical profiles. For example, in the Sotrovimab trial, it was shown that in the placebo group 13 of the 21 patients that died, died due to covid19‐related pneumonia, which might have been linked to changes in NLR, CRP and potassium as shown here [23]. Data from our study would also help to potential monitor other experimental or repurposed therapeutics such as nafamostat mesylate [24, 25]. For example, In four consecutive SARS‐Cov‐2 positive critically ill patients, administration of nafamostat mesylate was associated with hyperkaliaemia. We have identified hyperkaliaemia as a risk factor for death in this study [26, 27], potentially highlighting that this therapeutic might not offer the same level of protection as the others mentioned here.

## CONFLICT OF INTEREST

The authors have no competing interests.

## AUTHOR CONTRIBUTIONS

AR, TH‐S, WW and MSD designed the research study. AR collected data from the ICE system. AR, TH‐S, WW and MSD analysed the data. AR and MSD wrote the paper.

## ETHICS STATEMENT

The studies were conducted in accordance with the ethical principles of Good Clinical Practice. The data were extracted, anonymised and supplied by Bedfordshire Hospitals NHS Foundation Trust in accordance with internal information governance review. Ethical approval for this study was approved by the Independent Ethics Committee at Coventry University.

## Data Availability

The data that support the findings of this study are available on request from the corresponding author. The data are not publicly available due to privacy or ethical restrictions.
